# Reduced re-infection rates with postoperative oral antibiotics after two-stage revision hip arthroplasty

**DOI:** 10.1186/1471-2474-14-123

**Published:** 2013-04-05

**Authors:** Aaron J Johnson, Michael G Zywiel, Lynne C Jones, Ronald E Delanois, D Alex Stroh, Michael A Mont

**Affiliations:** 1 Rubin Institute for Advanced Orthopedics, Sinai Hospital of Baltimore, 2401 West Belvedere Avenue, Baltimore, MD, USA; 2 Johns Hopkins Orthopaedics at Good Samaritan Hospital, 5601 Loch Raven Boulevard, Baltimore, MD, USA

**Keywords:** Peri-prosthetic infection, THA, Total hip arthroplasty, Antibiotic, Re-infection rates

## Abstract

**Background:**

Surgeons are often trying to decreased reinfection rates following two-stage reimplantation arthroplasty, which range from 3.2% to 13% because multiple staged revision procedures for infection can be costly and have high morbidity. We therefore asked: (1) Did the use of postoperative oral antibiotics reduce reinfection rates after 2-staged revision of THA? And (2) how did this compare with the infection rate after aseptic revision procedures?

**Methods:**

We identified all patients who underwent two-stage revision THA for a periprosthetic deep hip infection and found 66 patients (67 hips) who had a minimum 24 months’ followup. Twenty-two of the 66 procedures (33%) were followed by a minimum of 14 days of postoperative oral antibiotics (mean, 36 days; range, 14 days to lifelong), while 44 were prescribed only immediate parenteral postoperative antibiotic therapy (mean, 1.3 days; range, 1–3 days). We then identified 407 patients (410 hips) who underwent aseptic revision hip arthroplasty and evaluated the infection rate in these patients for comparison; these patients were treated with 24 hours of postoperative parenteral antibiotics. The authors used previously described creteria to establish the presence of infection.

**Results:**

There were no reinfections in the group receiving oral postoperative antibiotics compared to six reinfections (13.6%) in the 44 patients not receiving oral antibiotics. We observed infection in 2 of the 410 hips (0.5%) revised for aseptic reasons.

**Conclusions:**

We believe that our findings warrant further investigation for using postoperative oral antibiotics after reimplantation for periprosthetic infection in an effort to decrease the likelihood and risks associated with additional revision arthroplasty procedures.

## Background

Deep infection after THA is a devastating complication that poses a challenge for the orthopaedic surgeon in terms of the best treatment strategy [1-4]. Treatment methods include multiple irrigation and débridement with implant retention, single-stage revision, two-stage revision, and long-term antibiotic suppression therapy [1-8]. Treatment methods may depend on the type of infection, surgeon preference, patients’ medical comorbidities, and overall health status. However, the most common technique to control deep infections is staged reimplantation of the prosthetic components after a period of typically intravenous antibiotic therapy.

Although the overall infection rate for primary THA is reportedly less than 1% [4,5,7,8], the reinfection rates after two-stage reimplantation arthroplasty range from 3.2% to 13% [9-13]. The recent AAOS guidelines have highlighted recent advances in our ability to diagnose and recognize infections pre-operatively. However, a number of authors have questioned the optimal duration of parenteral antibiotics during staged revision arthroplasties [14-17]. Due to this, there is currently no definitive “gold standard” consensus regarding (1) whether post-reimplantation antibiotics are necessary, and (2) if they are used, are PO antibiotics effective in reducing re-infection rates.

Interestingly, at the authors’ institution over the past 10 years, we noticed that some patients had been treated with longer-duration postoperative oral antibiotics after two-stage reimplantation (minimum of 2 weeks), while others had not been treated for more than 1 to 3 days. We therefore asked: (1) Did the use of postoperative oral antibiotics reduce re-infection rates after revision THA due to periprosthetic joint infection? And (2) how did this compare with the infection rate after aseptic revision procedures?

## Methods

We reviewed the database of all patients treated at our center to identify those patients treated with a two-stage revision of a THA for a periprosthetic infection, as well as for those patients who underwent revision for aseptic loosening and had a minimum of 24 months’ followup. Between 2000 and 2007, 508 revision THA procedures were performed. Of those, 410 revisions were performed on 407 patients for aseptic causes, and the remaining 98 revisions, in 96 patients, were for a periprosthetic hip infection. Of those, 31 periprosthetic infections were treated with irrigation and débridement or single-stage revision THA. The remaining 67 hips in 66 patients underwent two-stage revision of their periprosthetic joint infection. Thus, all patients were treated with initial explantation of components, interim antibiotic spacer placement with concomitant intravenous antibiotics for a minimum of 6 weeks, followed by re-implantation if there were no continued signs of infection.

All but one of these patients in this retrospective cohort study had late chronic infections; one patient developed an early postoperative infection with a draining sinus tract. In three cases, the infected index THA had been implanted at the authors’ center, while the remaining cases were referred from outside institutions. All cases received a minimum of 24 hours of parenteral perioperative antibiotics.

All patients were operated on by one of two senior authors (MAM, RED) using the same surgical approach performed during the primary arthroplasty procedure. For initial explantation procedures, after gaining exposure of the joint, specimens were obtained from femoral tissue, acetabular tissue, capsular tissue, and any other suspicious areas and were sent for intraoperative frozen analysis for signs of acute inflammation as described below, permanent formalin pathologic analysis for comparison to intraoperative frozen sections, and microbiologic culture on blood agar plates for a minimum of 120 hours. Multiple frozen sections were again taken intraoperatively from the same locations (i.e. the femur, acetabulum, and any suspicious-appearing areas) at the time of the second-stage procedure, and the final prosthetic components were not reimplanted unless the histologic evaluation confirmed no signs of acute inflammation. No patients were implanted who had positive intra-operative frozen sections, and none of the re-implanted patients grew positive cultures.

The 66 patients were divided into two cohorts: Group I was made up of 22 (23 hips) of the 66 patients who received a minimum of 14 days of p006Fstoperative oral antibiotic therapy (mean, 36 days; median, 42 days; range 14 days to lifelong) after reimplantation of components and were identified as cases; the remaining 44 patients (Group II) did not receive more than 3 days of postoperative antibiotics (mean, 1.3 days; range, 1–3 days) and were used as a comparison group. The primary diagnoses and indications for the initial THA were comparable between the two groups (Table 1). We identified numerous organisms on culture at the time of initial explantation, with methicillin-resistant Staphylococcus aureus (MRSA) the most prevalent in both groups (Table 2) (31% in the oral antibiotic group compared to 25% in the group without oral antibiotics [p = 0.5]).

**Table 1:** Primary diagnoses and demographic variables

	**Patients receiving postoperative oral antibiotics**	**Patients not receiving postoperative oral antibiotics**	**p-value**	**Patients undergoing aseptic revision**
Number of patients (hips)	22 (23)	44 (44)		407 (410)
Men	12 (12)	17 (17)	0.30	162
Women	10 (11)	27 (27)	0.30	248
Primary diagnosis				
Osteoarthritis	14	27	1.0	313
Osteonecrosis	3	7	1.0	58
Developmental hip dysplasia	1	4	0.66	4
Trauma	3	5	1.0	13
Rheumatoid arthritis	0	2	0.54	22
Gaucher disease	1	0	0.33	0
Number of patients at increased infection risk	11 (50%)	21 (48%)	1.0	244 (60%)
Age (years)^*^	58 (26–87)	57 (19–89)	1.0	60 (16–90)
Body mass index^*^	27.3 (17.1–47.9)	30.5 (18.4–56.1)	0.13	30.8 (16.8–53.3)

^*^Values are expressed as means, with ranges in parentheses.

**Table 2:** Cultured organisms and infection categorization

**Organism**	**Hips of patients receiving postoperative oral antibiotics**	**Hips of patients receiving only perioperative antibiotics**
	**(Group I)**	**(Group II)**
Gram positive		
Methicillin-resistant Staphylococcus aureus	10	15
Coagulase-negative Staphylococcus	2	9
Enterococcus	3	2
Methicillin-sensitive Staphylococcus aureus	1	4
Streptococcus viridans	2	0
Vancomycin-resistant Enterococcus	2	0
Corynebacterium	1	1
Streptococcus, Group A	0	1
Streptococcus, Group B (agalactiae)	0	1
Streptococcus, Group F	1	0
Gram negative		
Pseudomonas	1	5
Enterobacter	1	4
Escherichia coli	2	1
Klebsiella	1	2
Acinetobacter	1	2
Proteus	1	1
Serratia	0	2
Bacteroides	1	0
Citrobacter	0	1
Morganella	0	1
Negative cultures	2	10
Reinfection rates by number of infecting organisms		
Polymicrobial (Re-infection)	8 (0)	16 (3)
Single microbial (Re-infection)	13 (0)	18 (1)
Culture negative (Re-infection)	2 (0)	10 (2)

The mean age of patients at time of two-stage revision was 58 years (range, 27–87 years) in the group that received postoperative oral antibiotic therapy and 57 years (range, 19–89 years) in the group that did not. The minimum follow-up for both groups was 24 months (mean, 45 months; range, 24–105 months), with no patients lost to follow-up. A third group (Group III) used for comparison was the patients who underwent revision hip arthroplasty for aseptic reasons (407 patients, 410 hips), who had a mean age of 60 years (range, 16–90 years) (Table 1). Institutional review board approval was granted for this study by the LifeBridge Health Institutional Review Board (Reference Number 1998).

Demographics and comorbidities were compared between the two cohorts of patients who underwent revision for periprosthetic infection to evaluate for potentially confounding variables, including risk factors for periprosthetic infection (such as prolonged operative time, multiple prior surgeries) or immunosuppressive comorbidities that might predispose to infection (diabetes mellitus, rheumatoid arthritis, history of cancer or other chronic disease requiring immunosuppressive treatment, or other diseases that result in an inability of the patient to mount a sufficient immune response). Eleven of the 22 patients in Group I had one or more of the above risk factors (50%), as did 21 of the 44 (48%) patients who did not receive antibiotics (p = 1.0).

All patients were re-implanted only after they had completed a 6-week course of intravenous antibiotics during a period of antibiotic cement spacer placement, as well as no positive intra-operative frozen sections for acute inflammation. The decision to give longer-duration oral antibiotics after reimplantation arthroplasty was made by the consulting infectious disease physician, but was not based on a predetermined algorithm. Additionally, there was no preselected assignment of patients based on any patient factors. At the senior author’s present institution, there was no protocol in place for prescribing oral antibiotics after revision THA. Upon review of our database of patient information, we noted that some patients had been prescribed oral antibiotics upon discharge, while others had not. When oral antibiotics were given after the reimplantation procedure, they were started within 48 hours of the procedure and continued for a minimum of 14 days. When cultures from the first-stage procedures were positive, the susceptibilities from these cultures were used to guide therapy; otherwise, treatment was empirical, based on the most likely pathogen.

Periprosthetic infections were diagnosed using the criteria of Leone and Hanssen [18], modified for the hip. Any one of the following four criteria was sufficient for the diagnosis of a periprosthetic infection: (1) two or more positive cultures with the same organism; (2) histologic evidence of an acute inflammatory response seen on intraoperative frozen section, which was defined by the authors as a mean of greater than 10 polymorphonucleocytes observed on intraoperative frozen histologic section was considered positive for infection based on the criteria used by multiple other studies [19-21]; (3) gross purulence; or (4) a draining sinus tract that communicated with the joint space.

Following reimplantation procedures, patients were followed at 1 week, 3 weeks, 3 months, 6 months, and annually for a minimum of two years. At all visits up to and including the 6-month visit, the patients were seen and evaluated with a thorough history and physical exam, including inspection of the incision for any signs of erythema, edema, pain, dehiscence, or a draining sinus tract. Antero-posterior and lateral radiographs were performed at each follow-up visit. Each radiograph was examined by the senior author for appropriate component placement and for any signs of radiolucencies or subsidence. Additionally, if there were any clinical signs of re-infection (as described above) laboratory analyses were performed, which included, but were not limited to: complete blood count with differential, basic metabolic panel, liver function tests, Westergren sedimentation rate, and C-reactive protein. For the purposes of this study, re-infection of the two-stage revision THA was defined as a subsequent deep infection of the hip prosthesis requiring operative treatment, with the same criteria used as was previously described for the presence of a periprosthetic infection. Seromas and superficial wound infections were not considered failures unless they extended through the deep fascial layers or to the hip prosthesis.

Data were extracted and collated using Excel® spreadsheet software (Microsoft Corp, Redmond, WA). Success rates were compared between the group of patients who received postoperative oral antibiotics and those who did not using a Fisher’s exact test by testing the null hypothesis that the number of reinfections was evenly distributed between the two groups. Although there appeared to be no demographic differences between the two groups, there remained a possibility of bias due to nonrandom selection. Because of this, a binomial proportion confidence interval was used to determine the 90% confidence intervals (CIs) for reinfection rates in these two groups. We then compared the incidence of reinfection between the group of patients who were not treated with postoperative oral antibiotics and those who underwent aseptic revision THA at the authors’ institution over the same time period again using a Fisher’s exact test, with the same null hypothesis as above, as well as providing 90% binomial proportion CIs.

## Results

The incidence of reinfection was greater (p = 0.087) in Group II than in Group I. There were no reinfections in Group I, nor were there complications due to the administration of antibiotics. Six of 44 patients (13.6%) in Group II were reinfected (Table 3).

**Table 3:** Characteristics of patients experiencing reinfection after two-stage revision THA

**Patient**	**Primary diagnosis**	**Original organism**	**Reinfection organism**	**Time to reinfection (weeks)**	**Outcome**
1	Osteomyelitis	MRSA, Serratia	MRSA	36	Repeat two-stage revision THA
2	Hip trauma	Negative	Pseudomonas	60	Repeat two-stage revision THA
3	Osteoarthritis	MRSA	MRSA	1	Retention of components THA
4	Osteoarthritis	Enterococcus, Pseudomonas, MRSA	Enterococcus, E Coli, Pseudomonas, MRSA	2	Femoral prosthesis, revision THA and TKA
5	Osteoarthritis	MRSA, Proteus	MRSA	7	Resection arthroplasty
6	Osteonecrosis	Negative	Negative	1.5	Single-stage revision

MRSA = methicillin-resistant Staphylococcus aureus.

When compared to the patients who underwent aseptic revision THA, the group that did not receive oral antibiotics experienced a higher (p < 0.001) infection rate. In the group of 410 hips that underwent aseptic revision, only two were subsequently infected. Both patients presented within 30 days of their revision arthroplasty.

Of the six patients who developed a reinfection after two-stage revision THA, two underwent a repeat two-stage revision procedure, one underwent irrigation and débridement with retention of the implant, one underwent a repeat staged revision with a total femoral prosthesis, one underwent a resection hip arthroplasty, and one underwent a single-stage hip revision with reimplantation of new components. All cultured organisms are reported (Table 2). Three patients (Patients 1, 4, and 5 in Table 3) were reinfected with the same organism, all of which originally were polymicrobial (including MRSA), and subsequently grew MRSA after they underwent two-stage revision. One patient (Patient 3 in Table 3) grew MRSA in culture before and after the staged revision procedure, and the remaining two patients (Patients 2 and 6 in Table 3) never had a positive culture during their initial revision procedures.

## Discussion

While the risk of infection after primary THA has been reduced to approximately 1% in most cases [4,5,7,8], there remains a substantial percentage of patients who became reinfected after two-stage reimplantation. These reinfections can be extremely difficult to eradicate, costing in excess of $100,000 per revision [22]. Because of this, it is important to look for simple, cost-effective, and straightforward methods to improve patient outcomes and reduce the rates of reinfection after two-stage revision THA. After the senior author had moved to his current institution, he noticed an increase in infection rates, which prompted further inquiry into the oral antibiotic regimen being prescribed by the various infectious disease consultants. This led to the discovery that some patients had received postoperative oral antibiotics while others had not and prompted the present investigation.

Antibiotic use during two-stage revision arthroplasty has been discussed regarding duration of treatment, specific antimicrobial agents, and their effect on specific organisms (eg, MRSA). The appropriate duration of postoperative parental antibiotics has been a topic of contention [15,16]. In a study of 31 consecutive infected THAs, McKenna et al. [16] reported no reinfections and concluded a shortened postoperative course of parenteral antibiotics was successful in treating infection. Bassetti et al. [14] reviewed the duration of parenteral versus oral linezolid and found both were effective in orthopaedic procedures. Additionally, Oussedik et al. [17] concluded linezolid is effective for the oral treatment of infected joint arthroplasties. Other authors have noted greater difficulty when treating periprosthetic hip infections when drug-resistant pathogenic organisms, such as MRSA, are identified [6,14,23,24]. In 2008, Pulido et al. [24] reported on 66 drug-resistant periprosthetic infections with either MRSA or MRSE infections with 32 undergoing two-stage revisions. Only 24 of these 32 patients had their infection eradicated (75% success rate). In our study, MRSA infections were the most common infection in both cohorts (those receiving and those not receiving postoperative antibiotics). In contrast to the findings by Pulido et al. [24], the patients who received postoperative oral antibiotics had no reinfections. This suggests oral antibiotics after reimplantation THA may improve reinfection outcomes when treating drug-resistant organisms.

The infection rate in this report after aseptic hip revisions was 0.5% and is consistent with that reported in the literature [4,5,7,8]. Previously reported success rates for two-stage revisions range from 86% to 94%, which are consistent with our findings (Table 4) [5,23,25-28]. The cohort that received postoperative oral antibiotics had no reinfections, which is comparable to the low infection rates seen by some authors in primary arthroplasty procedures [4,5,7,8]. In contrast, the cohort that did not receive postoperative oral antibiotics had a higher infection rate than the aseptic revision cohort, with the reinfection rate comparable to the reports in the literature of higher reinfection rates after two-stage revision for infected THAs [5,23,25-28]. It is uncertain what the mechanism is that leads to this difference in re-infection rates. The authors postulate that these oral antibiotics may have prevented biofilm formation on the new implants, or may have provided further bacteriocidal effects the further eliminate any remaining bacterial load. Although our sampling for acute inflammation on frozen pathologic sections has low false negative rates [29,30], there is still variability in the quality of pathology departments and their ability to identify retained signs of inflammation.

**Table 4:** Reported success rates of two-stage revision arthroplasty

**Study**	**Year**	**Number of hips**	**Success rate (%)**
Garvin and Hanssen [14]	1995	423	91
Cierny and DiPasquale [7]	2002	43	88
Ammon and Stockley [1]	2004	57	86
Hoad-Reddick et al. [16]	2005	53	89
Cordero-Ampuero et al. [8]	2007	16	94
Stockley et al. [29]	2008	114	88
Present study	2010		
No oral antibiotics		44	86
Oral antibiotics		23	100

There were several limitations of this study. This was an observational study and there was no preselected assignment of patients based upon any demographic factors. While we recognize these two groups were not randomized, we believe the two groups had comparable demographic data, primary diagnoses, and distributions of infecting organisms. Next is the concern regarding the power of the study, given the small sample size. A retrospective power analysis determined the study had 91% power and the ability to detect a 7% difference in infection rates. With minimal differences in confounders, there still remains the possibility that the two cohorts may have had a selection bias, which could have skewed the results. We believe that the study was sufficiently powered to make the preliminary conclusion that oral antibiotics after two-stage revision THA may reduce periprosthetic hip reinfection rates in the hopes of encouraging further, more rigorous studies of this nature in the future. Additionally, it is always difficult to determine the exact pathogenesis of the repeat infection: i.e. was the infection due to a recurrence of the original pathogen, or is the infection a new event, independent of previous treatments. Furthermore, prolonged courses of antibiotics may have an improved ability to eradicate remnant pathogens that were not originally detected at the re-implantation procedure. The emergence of drug resistant organisms is always a concern when prescribing antibiotics, even though all patients who are undergoing 2-stage revision arthroplasty have already been treated with a minimum of 6 weeks of IV antibiotics. This was made increasingly difficult based on the number of patients with negative cultures. This may be due to a lack of cultures prior to the initiation of antibiotics; frequently, primary care physicians or emergency department staff will start patients on antimicrobial therapy prior to attaining cultures. In future studies, care should be taken to ensure that cultures are taken prior to the initiation of antibiotic therapy. Additionally, we did not address the effect of duration of antibiotic treatment (i.e. 2 weeks of antibiotic therapy may be comparably effective in reducing re-infection rates as the median 6 weeks reported in the present study). Future prospective, randomized studies could possibly evaluate the efficacy of different durations of therapy; however, the power needed for such a study may be prohibitively small to detect a difference given the positive results reported here with 6 weeks of treatment. Many factors, including the virulence of the infecting organism, antibiotic mechanisms, patient-specific factors (such as immunosuppression), and specific organism-antibiotic susceptibilities can influence the required duration of antibiotics. In future work, many of these factors should be rigorously addressed in the form of a prospective randomized trial to determine the optimal treatment type and time.

## Conclusions

In conclusion, we have had success in reducing recurrent infections after two-stage revision for infected THAs by using a mean of 36 days of postoperative oral antibiotics. The re-operation rates for subsequent infection among patients who received two-stage hip revisions without longer-duration postoperative oral antibiotics was consistent with the literature at approximately 14%. Although the difference was not statistically significant, over a 7-year period, not a single patient who received postoperative oral antibiotics after staged revision went on to require further surgery for infection after reimplantation. We emphasize that the risks and benefits of using broader-spectrum antibiotics must always be evaluated and patients on such protocols should be under the care of an experienced infectious disease specialist. In conclusion, we put forth this preliminary work in the hope it will encourage larger, multicenter, prospective studies to help determine the most appropriate pharmacologic therapy and treatment duration.

## Competing interests

No external funding was received specifically in support of this work.

## Authors’ contributions

AJJ collected data, participated in data analysis, and drafted the initial manuscript; MGZ assisted with data collection and analysis, statistical analysis, and manuscript editing; LCJ participated in study design, statistical methods, and manuscript editing; RED was one of the senior authors, contributed patients, assisted with data analysis, and study design; DAS assisted with data collection and analysis; MAM provided initial study design, contributed patients, contributed to experimental design, assisted with manuscript drafting, and final manuscript editing. All authors read and approved the final manuscript.

## Pre-publication history

The pre-publication history for this paper can be accessed here:

http://www.biomedcentral.com/1471-2474/14/123/prepub
